# The Care of Rheumatic Children

**Published:** 1913-02-01

**Authors:** C. O. Hawthorne


*To the Editor of* The Hospital.

### The Care of Rheumatic Children.

*To the Editor of* The Hospital.

Sir,—I think I may venture to claim a special personal interest in Dr. Poynton's valuable and vigorous lecture, published in your issue of January 18. For several years and on repeated occasions I have urged that a sustained and systematic effort should be made by the medical profession to prevent the occurrence of heart disease in childhood; in particular I may refer to a discussion on the subject at the annual meeting of the British Medical Association in 1901, to an article which appeared in the *Polyclinic* in February 1902, and to a paper which I read to the Society for the Study of Diseases in Children in 1907.

On each of these occasions I have suggested that the reason why cardiac disease complicates the rheumatism of childhood so much more frequently than it does the rheumatism of the adult is to be found in the contrasted forms in which the disease expresses itself, and in the different relations which these hold to the practice of rest. In the rheumatic fever of the adult the painful and swollen joints make early and complete rest imperative; the heart is therefore placed under the conditions most likely to avoid disaster. But in the rheumatism of the child the joints largely escape, and consequently there is no element compelling the rest which the adult must needs endure; the heart, in other words, is not protected, and, as is to be expected in such circumstances, it frequently suffers. Whether this is or is not the complete explanation, there can be but little doubt that it is a part of the truth. Hence the practical problem would appear to be to secure for the child the protective rest which in the case of the adult the disease itself imposes. But no such end is possible until the parents or guardians of rheumatic children having a rheumatic inheritance are made to understand that the apparently slight illnesses of these children are really rheumatism, and are likely to be attended by heart disease; and, further, that to prevent such a catastrophe early and complete rest affords the best security.

Now this teaching to be effective must be conveyed at the earliest possible date in the family history, and for practical purposes this means the first detection of a rheumatic inheritance in the family record. Let patients and guardians be warned in time, and there is some chance of securing for the children the prompt and complete rest which is necessary for their protection. In other words, the contention which I have repeatedly ventured to advance is that when a practitioner detects in a patient evidences of rheumatism, his duty is not confined to the individual under his immediate care; on the contrary, it extends, for preventive purposes, to the other members of the family, and more especially to those members who on account of their youth are particularly prone to the less overt but most dangerous forms of the disease. Were this claim recognised as an imperative duty by the profession generally, is it not in the highest degree likely that at least some children would be saved from the disaster of organic heart disease? It is, of course, excellent that children who have already suffered this disaster should be effectively cared for and skilfully treated. But still better is it to prevent further disasters of the like order. Let a single rheumatic event in the family circle be the occasion for the warnings above described, and the profession will at least have acted to the extent of its knowledge. What the future holds in the way of prevention or cure we know not. But we certainly know (1) that the children of rheumatic parents are themselves apt to suffer from rheumatism; (2) that in these children rheumatic disease generally takes forms which are not recognised popularly as "rheumatism"; (3) that all such manifestations are liable to be accompanied by organic disease; and (4) that the best known protection against this complication is complete rest during the whole of the time that active evidences of rheumatism are present. If this information were systematically and as an ordinary professional obligation conveyed at the first available opportunity to all parents known to be rheumatic, these would be forewarned of the dangers attending the apparently slight illness of their children. And if they acted on this warning—as doubtless in many instances they would—is it not highly probable that the terrible story of heart disease in children as it is read to-day in all our experiences would, at least, in some measure—and perhaps in a substantial measure—be mitigated and redeemed?—I am, Sir, yours faithfully,

C. O. Hawthorne.

